# Editorial: Using case study and narrative pedagogy to guide students through the process of science

**DOI:** 10.3389/fmicb.2024.1490868

**Published:** 2024-10-14

**Authors:** Davida S. Smyth, Pat Marsteller, Courtney Carroll Alexander, Alex L. Deal, Carlos C. Goller

**Affiliations:** ^1^Department of Natural Sciences, Texas A & M University-San Antonio, San Antonio, TX, United States; ^2^Department of Biology, Emory University, Atlanta, GA, United States; ^3^Department of Biology, University of North Carolina at Pembroke, Pembroke, NC, United States; ^4^Department of Translational Neuroscience, School of Medicine, Wake Forest University, Winston-Salem, NC, United States; ^5^Department of Biological Sciences, North Carolina State University, Raleigh, NC, United States

**Keywords:** case study, molecular case study, narrative, literacy, scientific communication, R coding, underrepresented minority (URM) students, diversity

Novel technologies and methods continue to propel discovery; however, the equipment and data needed to train new scholars are often inaccessible in higher education. Narratives describing the process of modern scientific discovery engage and empower students and instructors to learn new techniques and participate in inquiry. In this Research Topic, scholars share how they use the power of narrative to train students and inform the public.

In Froney et al. the authors use real data sets to introduce students to phenotypic screening and the statistics behind this method of drug discovery. The introduction to phenotypic screening and the statistical analyses supporting the method described the benefits to undergraduate and graduate students. Students work through a case study that follows a new graduate student in a cancer research lab as she runs a phenotypic screen. The case lays out the statistical analysis the graduate students would perform before giving students practice data sets to analyze themselves. The work can be done in Google Sheets, eliminating the need for costly programs. The case study was used in graduate and undergraduate classrooms and improved student confidence with quantitative skills, an especially difficult task for educators.

In Pomeroy et al. the authors present a case-study approach that exposes students to R programming in the context of high-throughput data collected using microarrays. The case study is relatable to students, involving the Lenski Lab's Long-Term Evolution Experiment (LTEE) and a newly hired graduate who has found a position at a biotech firm and been tasked with analyzing and visualizing changes in gene expression from 20,000 generations of the experiment despite having no R experience. The student takes a journey from the first steps in R analysis to practicing figure generation to manipulation and interpretation of large datasets. Modular in design, this case study can be adapted depending on the student's comfort level, and in testing, it was well-received by students and faculty alike. This case study directly responds to faculty who wish to expose students to R by encouraging an understanding of the utility of R rather than the rote production of code.

The article by Thomas et al. describes the development and implementation of a case study based on real-world human gut microbiome data to address issues proposed in a realistic fictional scenario. The case study follows the tale of Dr. Jones and her investigation into the relationship between early-life antibiotics and the gut microbiome. The authors tasked students with considering multiple aspects of the scientific process, such as properly identifying data to inform a research question, viewing and processing sequence datasets, analyzing data to align with the research question, and interpreting data to draw proper conclusions. The case study concludes with students presenting their findings either visually through an infographic or a written technical report, depending on the form of scientific communication that best fits with course goals. The authors describe tailoring this case study for both major and non-major courses and find that engagement with the case study led students, regardless of major, to draw meaningful conclusions about the importance of the human gut microbiome and the use of antibiotics in current society.

The Trujillo and Dutta article presents a detailed rationale and process for developing molecular cases that can engage biology, chemistry, and biochemistry students, and even the general public in understanding molecular structures and their applications in the real world. The article describes some of the work from the Molecular Case Network^1^, including a detailed example of one of the cases. The article describes the framework for developing and using MCSs at the intersection of technological, pedagogical, and content knowledge (TPCK), as well as their use in interdisciplinary teaching and learning. This community of educators has developed multiple cases across various topics that employ freely available molecular structures to engage students in exploring biological functions, macromolecular sequences, macromolecular structures, chemical environments, and molecular forces to explore structures and connect structure to function. The authors present one detailed example, “Happy Blue Baby,” to illustrate the points of their model. They also explore the rationale for using molecular stories to engage students and introduce them to bioinformatic resources. Since students and faculty often are unfamiliar with the tools, the MCN group created videos, tutorials, and other documentation to supplement the cases. The article also describes challenges when piloting cases and the mechanisms developed to overcome them.

While new technologies continue to evolve, case studies and narrative pedagogies offer the opportunity to engage students and help them practice their research skills while investigating new questions (Raza et al., 2020) (Figure 1). Case studies can be easily shared in an open education environment on the BioQUEST QUBES hub^2^, on the NSTA cases site^3^, and through several scientific society pages and journals.

**Figure 1:**
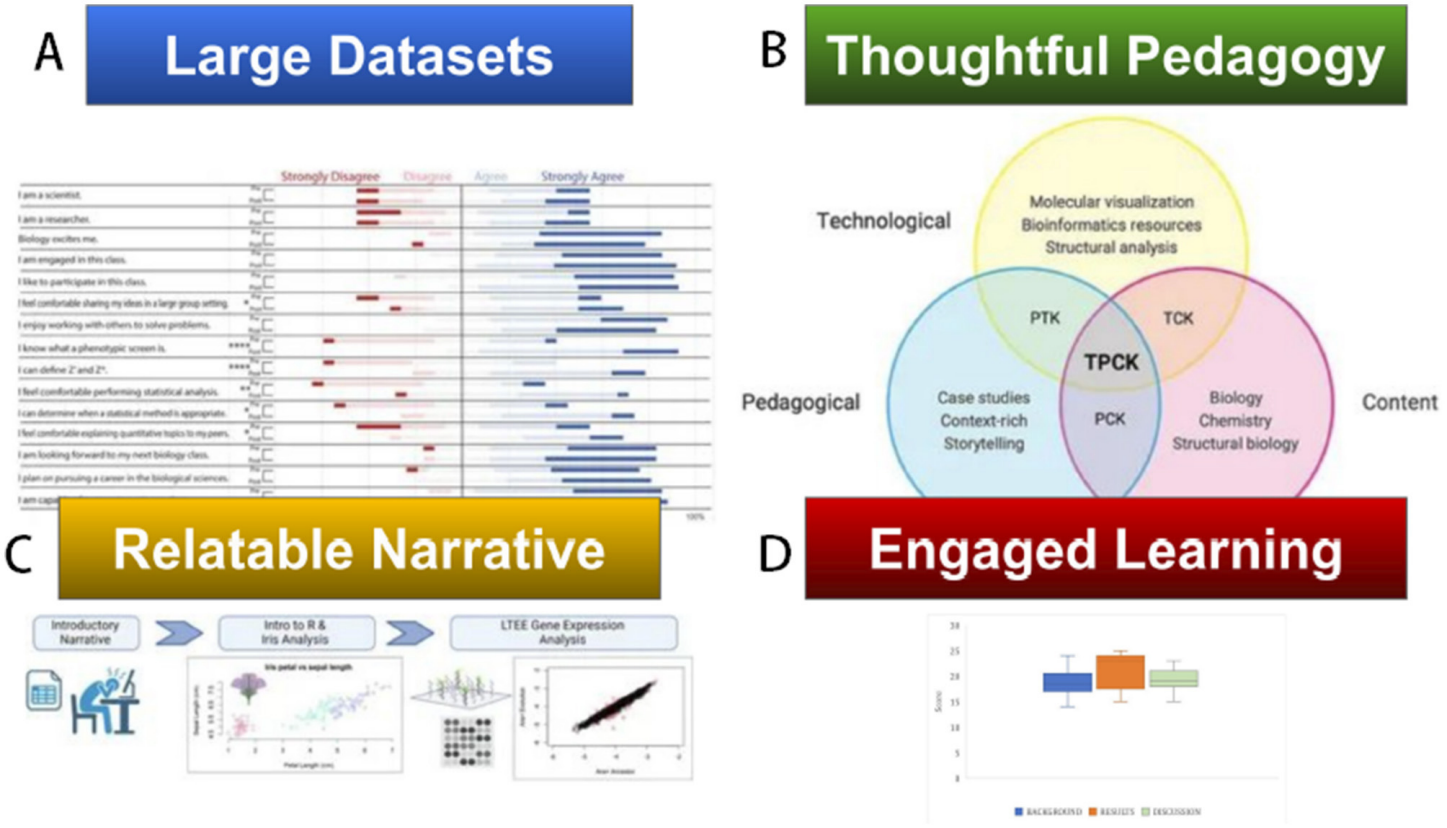
A captivating or challenging case study can engage learners in exercising important critical thinking and data analysis skills. Effective case study pedagogy requires thought-provoking, engaging, and relatable narratives. The interactions of these elements can result in increased learning, STEM engagement and co-creation of ideas and new directions with students and researchers. The images are from the corresponding publications. **(A)** Figure 3 from Froney et al. **(B)** Figure 1 from Trujillo and Dutta. **(C)** Figure 1 from Pomeroy et al. **(D)** Figure 2 from Thomas et al.

Stories and narratives can impact student engagement and have been demonstrated to improve learning (Bonney, 2015; Raza et al., 2020). Faculty development in using case studies in STEM, including development networks for new case studies and the support of hosting sites for OER resources for the long-term accessibility of these materials, should be a funding priority for agencies such as the National Science Foundation. We request that DBER faculty and education researchers help the community develop assessment instruments and design experiment studies to measure learning gains tied to case study style pedagogy. Data on impact will help us to encourage and convince all our colleagues to use and create their cases. Research into the impact of case studies on teaching the process of science, as well as the value of recognizing interdisciplinary perspectives while tackling real-world issues of relevance of STEM, is critical for our students and colleagues if we are all to be a part of the solution to wicked problems such as climate change, pandemics, and the impacts of emerging technologies. We hope you find these articles and exciting upcoming contributions of use and value and that you consider some of the approaches described for your classrooms.

## Footnotes

^1^ https://molecular-casenet.rcsb.org/

^2^ https://qubeshub.org/

^3^ https://www.nsta.org/case-studies
